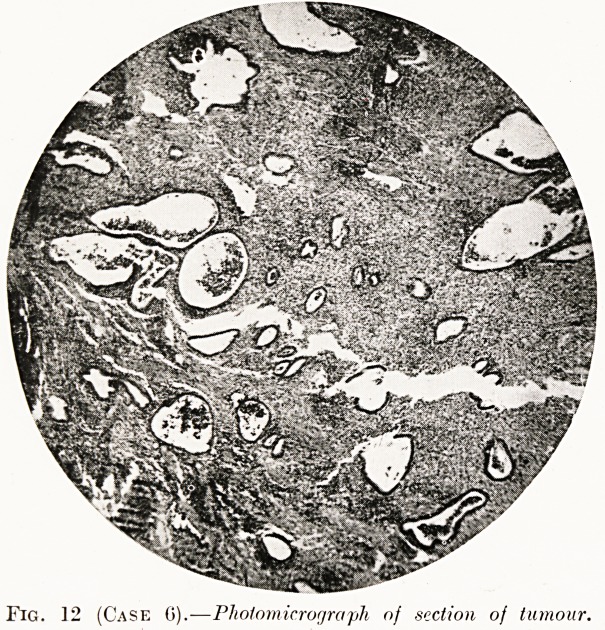# The Endometriomata

**Published:** 1927

**Authors:** A. D. Fraser

**Affiliations:** Pathologist, Bristol Royal Infirmary


### THE ENDOMETRIOMATA.

BY

A. D. Fraser, M.A., B.Sc., M.B.,

*Pathologist, Bristol Royal Infirmary.*

---

Endometriomata are tumours found exclusively in women between the age of twenty and the menopause. They occur in the uterus and Fallopian tubes, in the ovaries, the uterine and ovarian ligaments, the umbilicus, and in laparotomy scars.

Although microscopically benign, they are locally invasive and recurrence follows their incomplete removal. No authentic case of blood or lymph stream spread has yet been reported.

Since the earlier publications by Cullen[1] and by Sampson,[2] who both drew attention to the frequency of these growths, a very large number of cases have been reported by others, and they are now recognised to be a fairly common cause of female pelvic trouble. In one year Sampson[2] observed endometrial tumours in sixty-four out of three hundred and two abdominal operations for pelvic conditions.

The essential element of these growths is functioning endometrial tissue appearing as scattered islands in a matrix of fibrous tissue and smooth muscle. The latter is absent in some cases, particularly when the site is ovary. The islands of endometrium show the same structure as that of the normal uterus, glandular spaces lined by ciliated columnar cells and surrounded by the typical cytogenic stroma. The similarity is not only a histological one but is a functional one as well, for this ectopic endometrium goes through the menstrual cycle changes and also those of pregnancy and the menopause. Occasionally the glands communicate with a central cavity and the whole presents the appearance of a "miniature uterus."

Menstrual débris collects in the glands and cysts form containing material resembling soft chocolate. These "chocolate cysts" develop best in the ovary, where they may attain to a diameter of 5 centimetres.

No theory has as yet been evolved which can satisfactorily explain the occurrence of the tumours.

Müllerian or Wolffian rests may account for some, but not for those outside the uterus, tubes and ovaries.

Metaplasia of peritoneal epithelium following some form of irritation is an explanation that has now been discarded by most observers in favour of Sampson's implantation theory.

Sampson[2] believes that endometriomata of the ovaries originate through implantation of uterine mucosa cells, which have passed through the Fallopian tube, and that rupture of the chocolate cysts formed in ovaries leads to further implantations elsewhere in the peritoneum. Growths in laparotomy scars he considers to arise from soiling of the wound following incision into the uterus or handling of the tubes or ovaries. He explains those appearing in the uterine wall as being due to a direct invasion of the endometrium into a less resistant underlying musculature.

Sampson's views have received support from the experimental work on monkeys by Jacobson.[3] The latter has shown that pieces of uterine mucosa scattered in the pelvic cavity of the same animal can become implanted and give rise to endometrial growths.

Most observers, whilst admitting that a few of the

### PLATE IV.

FIG. 1 (CASE 2).—*Section of uterus showing circumscribed endometrioma. The two small encapsuled tumours are fibroids.*

FIG. 3 (CASE 3).—*Sagittal section of uterus showing diffuse endometrioma.*

FIG. 2 (CASE 2).—*Photomicrograph of section of tumour. Endometrial tissue embedded in smooth muscle.*

FIG. 4 (CASE 3).—*Photomicrograph of section of tumour. Some of the glands show early premenstrual change.*

tumours may originate in Müllerian rests, agree with Sampson.

*Clinical Signs and Symptoms.*—Dysmenorrhœa and menorrhagia are frequent accompaniments of these tumours, but do not serve to differentiate them from other pelvic conditions. When, however, the tumour is situated in the abdominal wall or inguinal region, there is, in addition, local pain and swelling and even external hæmorrhage during menstruation, which indicates very definitely the nature of the lesion.

The following cases observed during the last twelve months are illustrative of the condition. They have been grouped according to the classification suggested by Sampson :—

### DIRECT ENDOMETRIOMATA.

**Case 1.**—*Endometrioma of the Uterus.*—Miss E. G., æt. 51. Eighteen months menorrhagia with secondary anæmia. A large movable mass in the abdomen extending from symphysis pubis to near the umbilicus.

*Operation—Hysterectomy.*—A tumour the size of a small football was situated in the fundus of the uterus. On section it resembled an intramural fibroid, but was not encapsuled, and showed some hæmorrhagic areas. A provisional diagnosis was made of "fibroid become sarcomatous."

*Histological examination.*—A circumscribed endometrioma continuous with the normal endometrium. The stroma was hyperplastic smooth muscle. Some of the glands were cystic, but the majority were in the resting stage.

**Case 2.**—*Endometrioma of the Uterus.*—Mrs. S., æt. 48, multipara. Two years' menorrhagia with secondary anæmia.

*Operation—Hysterectomy.*—The fundus of the uterus near the left tube appeared swollen and suggested the presence of an intramural fibroid. Section showed a small growth rather resembling a fibroid, but not encapsuled (Fig. 1).

*Histological examination.*—A circumscribed endometrioma continuous with the normal endometrium. There was a stroma of hyperplastic smooth muscle. Most of the glands were in the resting stage (Fig. 2).

**Case 3.**—*Endometrioma of Uterus.*—Miss L., æt. 40. Twelve months' dysmenorrhœa and menorrhagia with secondary anæmia.

*Operation — Hysterectomy.* — The uterus was somewhat enlarged. On section the appearance was as shown in Fig. 3.

*Histological examination.*—A diffuse endometrioma in continuity with the normal endometrial layer, which was in the premenstrual stage. Most of the islands in the growth were at a similar stage, but some of the glands were cystic and contained blood and desquamated cells. The stroma was hyperplastic smooth muscle (Fig. 4).

### IMPLANTATION ENDOMETRIOMATA.

**Case 4.**—*Endometrioma of Ovary.*—Mrs. S. W., æt. 28. Married eighteen months. No children. Two years' history of dysmenorrhœa. On examination a somewhat tender lump was felt on the left side of the pelvis.

*Operation—Oophorectomy.*—The left ovary was adherent to the surrounding tissues. On section it was seen to contain one large chocolate cyst and several small ones (Fig. 5).

*Histological examination.*—Chocolate cysts of the ovary. The cysts were lined by columnar epithelium, and contained menstrual blood and desquamated cells (Figs. 6 and 7).

**Case 5.**—*Chocolate Cysts of the Ovaries and Endometrial Tissue in the Tubes and Recto-Vaginal Septum.*—Miss I. T., æt. 32. Two years' dysmenorrhœa and severe menorrhagia with attacks of abdominal pain during the last six weeks.

*Operation—Hysterectomy.*—Pelvic adhesions made the operation difficult. There were three large cysts each about 4 centimetres in diameter in the right ovary and two small ones in the left. They were full of soft brown coloured material. Similar smaller growths were present near the fimbriated ends of the tubes and in the recto-vaginal septum (Fig. 8).

*Histological examination.*—Endometrial tissue in the ovaries, tubes and recto-vaginal septum. No smooth muscle tissue observed. The cysts were lined by ciliated columnar cells and contained blood, desquamated cells and a few multinucleated giant cells (Figs. 9 and 10).

**Case 6.**—*Endometrioma of Umbilicus.*—Mrs. B. S., æt. 39. Married seventeen years, but never been pregnant. Six months ago a small painful swelling appeared at the umbilicus. It slowly increased in size, and was always more prominent during menstruation, which was now accompanied by severe pain in

### PLATE V

FIG. 5 (CASE 4).—*Cross section of ovary showing "chocolate cysts." The small notch at the top marks the site of perforation of the large cyst. The adhesions are not shown.*

FIG. 6 (CASE 4).—*Photomicrograph of section of ovarian tissue on the right of the large cyst, showing some of the smaller ones.*

FIG. 7 (CASE 4).—*Higher power view of area seen in Fig. 6.*

### PLATE VI.

Fig. 8 (Case 5).—*Sagittal section of uterus showing endometrioma of recto-vaginal septum penetrating into the cervix.*

Fig. 9 (Case 5).—*Photomicrograph of large cyst in cervix.*

Fig. 10 (Case 5).—*High power view of wall of cyst shown in Fig. 9.*

the lower abdomen and back. During the three last menstrual periods hæmorrhage occurred from a small blood blister which formed at the upper edge of the swelling. This blood blister ruptured usually on the last day of the menstrual flow and oozed blood for 24 hours, when it healed and disappeared, to reappear as the next menstruation approached.

*Operation—Excision.*—On section the growth appeared somewhat encapsuled with blood-stained areas and small cysts here and there (Fig. 11).

*Histological examination.*—Islands of endometrium embedded in fibrous and smooth muscle tissue. The glands were premenstrual in type and there was hæmorrhage into the cytogenic stroma (Fig. 12).

### TRANSPLANTATION ENDOMETRIOMATA.

**Case 7.**—*Endometrioma in Laparotomy Scar.*—Miss A. R., æt. 36. Five months ago a fibroid of the uterus was enucleated. The wound at one point never healed, and blood oozed from it during menstruation.

*Operation—Excision of Scar and Hysterectomy.*

*Histological examination.*—The scar contained islands of endometrium which extended from the peritoneum to the skin surface. The right ovary contained a chocolate cyst which was adherent to the posterior surface of the uterus. This cyst contained some multinucleated giant cells as well as blood and desquamated epithelial cells.

**Case 8.**—*Endometrioma in Operation Scar* (*Femoral Hernia*). —Miss C. C., æt. 44. Operation for right femoral hernia two years ago. Shortly after the wound healed a small painful swelling appeared just below the scar. It had been slowly increasing in size, and always became more swollen and painful during menstruation.

*Operation—Excision.*

*Histological examination.*—The cut surface showed cystic spaces full of blood. The growth was composed of endometrial islands in a fibrous tissue stroma.

**Case 9.**—*Endometrioma in Operation Scar* (*Appendicectomy and Oophorectomy*).—Miss K. W., æt. 25. Three years ago the appendix and one ovary was removed. The ovary had a blood cyst in it. About three months after healing of the operation wound there appeared in the scar a small swelling which gradually increased in size. There was no menstrual trouble.

On the contrary the dysmenorrhœa from which the patient had previously suffered had ceased since the operation. The swelling in the scar, however, was troublesome, becoming bluish coloured and painful a week after the menstrual flow had ceased, and remaining thus for about ten days.

*Operation—Excision.*

*Histological examination.*—Endometrial islands in a stroma of scar tissue.

I am much indebted to Dr. Geoffrey Hadfield who kindly made the photomicrographs.

REFERENCES.

[1] Cullen, *Arch. Surg.*, Chicago, 1920, i. 215.

[2] Sampson, *Idem*, 1921, iii. 245; *Am. J. Obst. and Gynec.*, St. Louis, 1922, 4, 451; *idem*, 1925, 10, 649; *idem*, 1926, 12, 459.

[3] Jacobson, *Archives Path. and Lab. Med.*, 1926, i. 169.

### PLATE VII.

FIG. 11 (CASE 6).—*Endometrioma of umbilicus.*

FIG. 12 (CASE 6).—*Photomicrograph of section of tumour.*

## Figures and Tables

**Fig. 1 (Case 2). f1:**
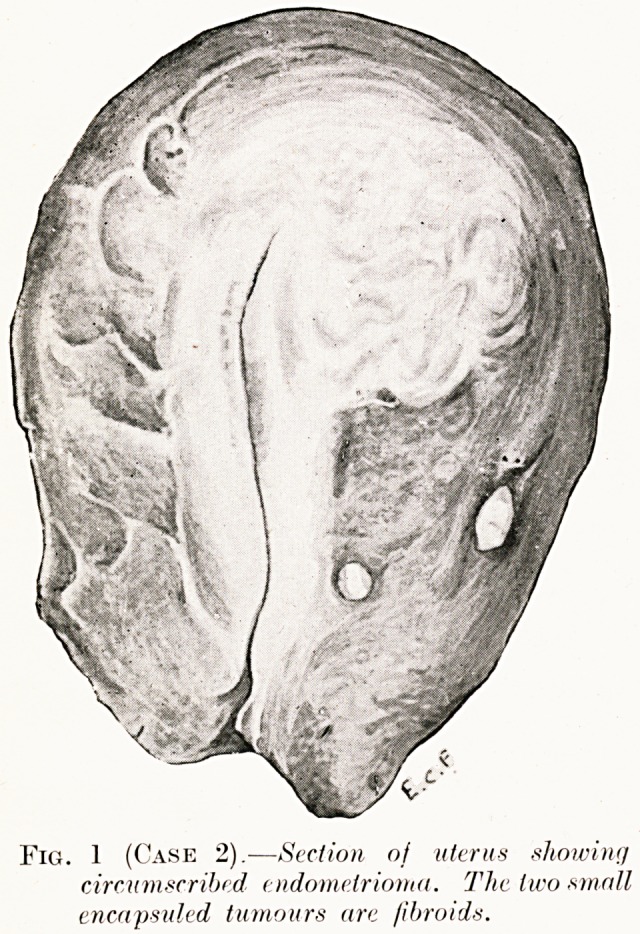


**Fig. 2 (Case 2). f2:**
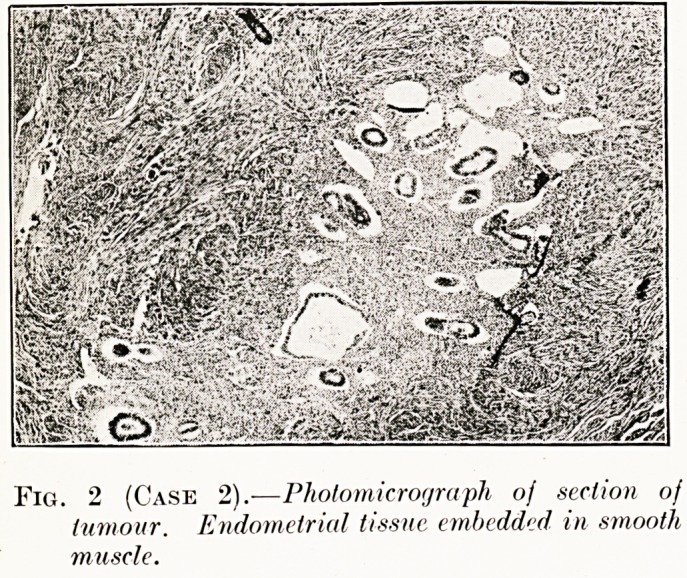


**Fig. 3 (Case 3). f3:**
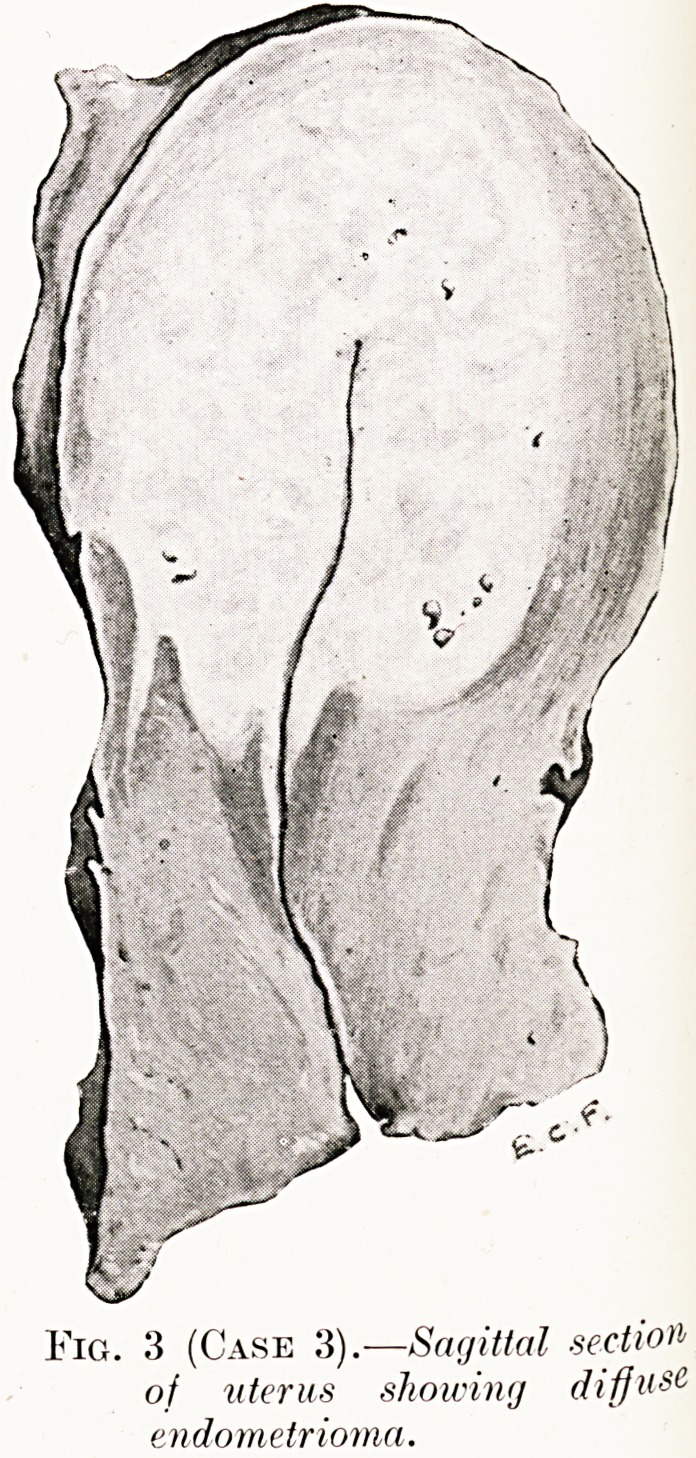


**Fig. 4 (Case 3). f4:**
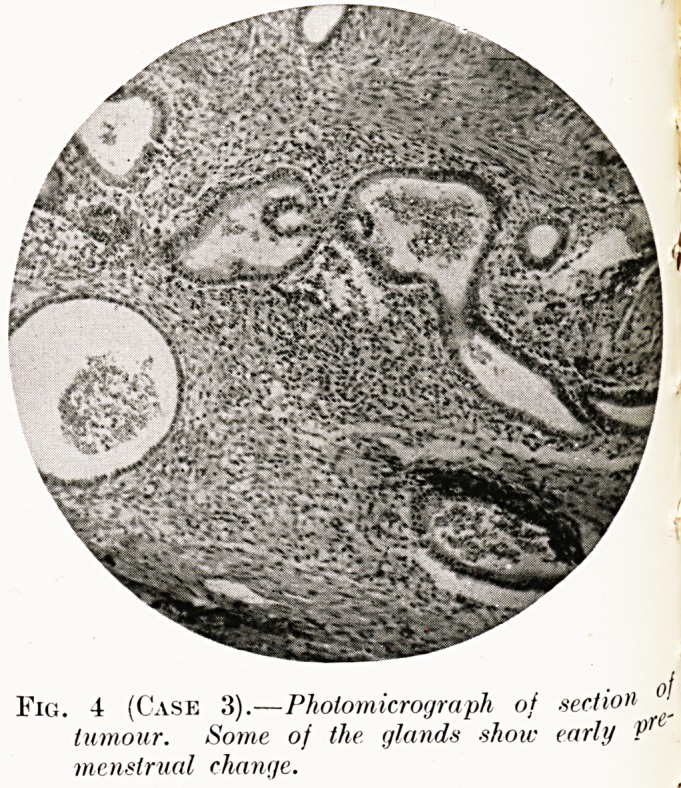


**Fig. 5 (Case 4). f5:**
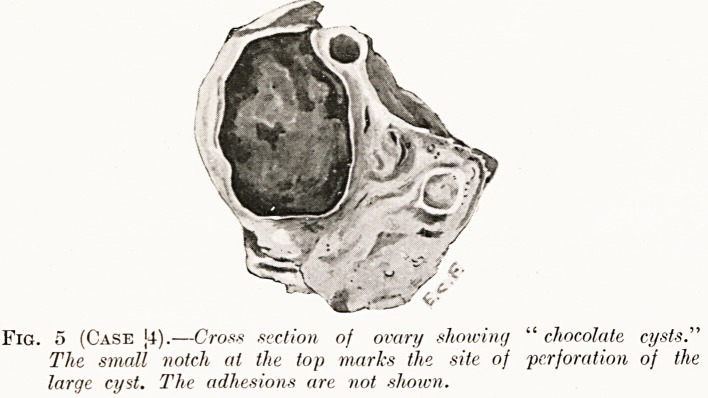


**Fig. 6 (Case 4). f6:**
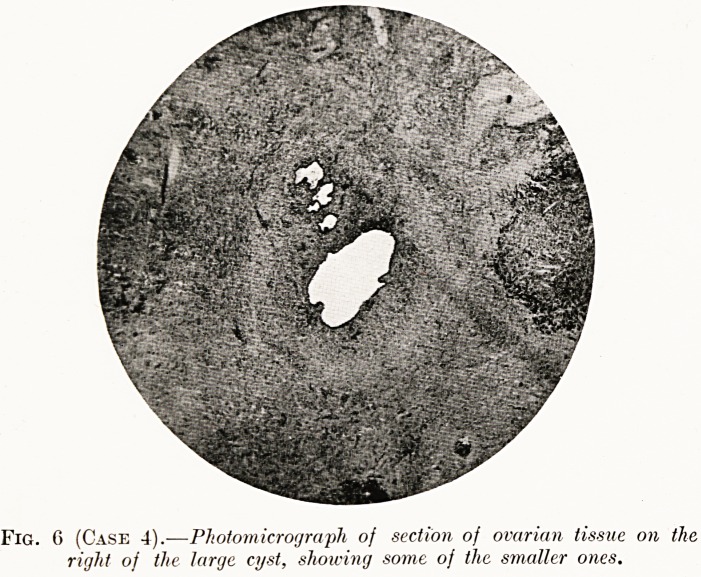


**Fig. 7 (Case 4). f7:**
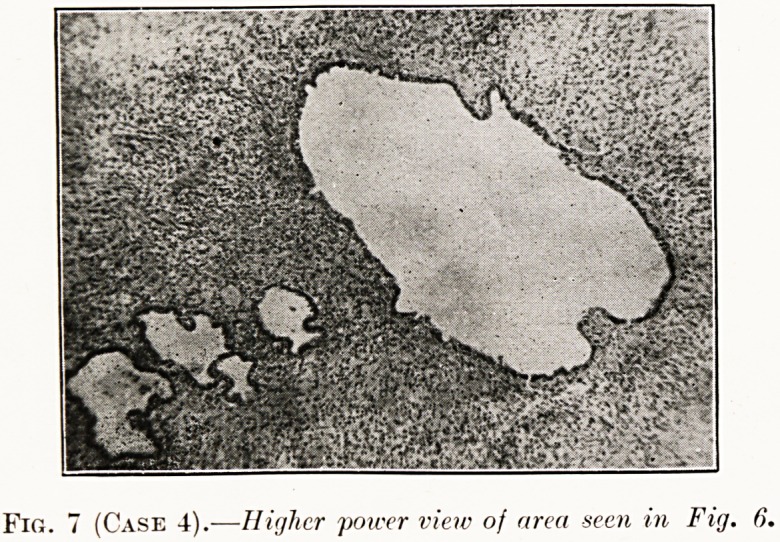


**Fig. 8 (Case 5). f8:**
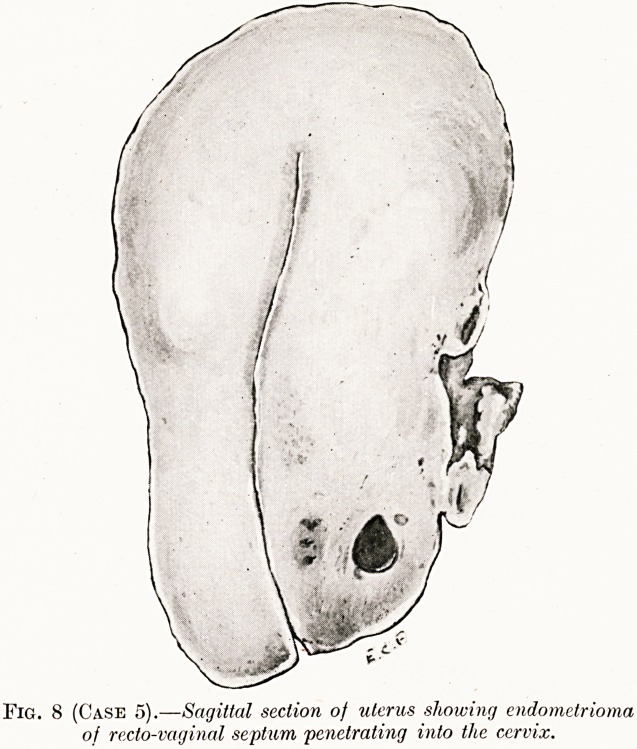


**Fig. 9 (Case 5). f9:**
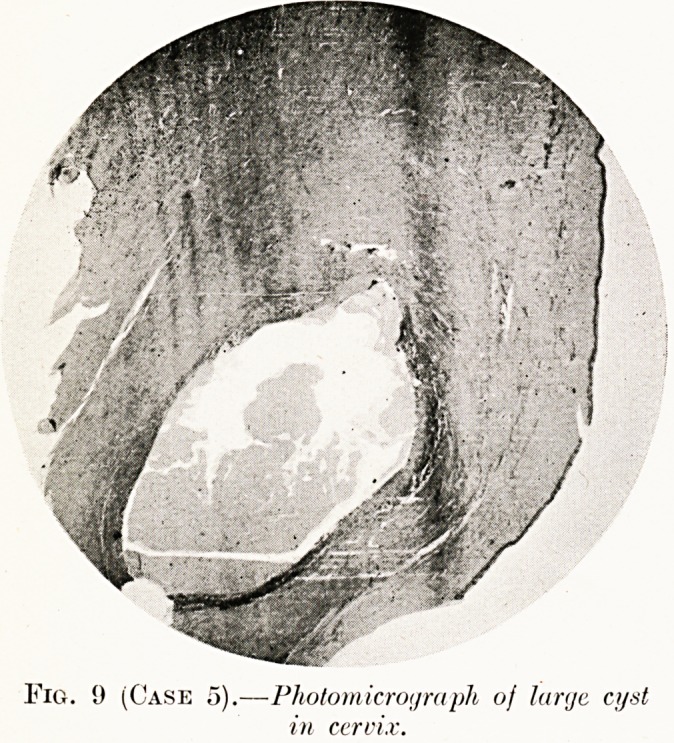


**Fig. 10 (Case 5). f10:**
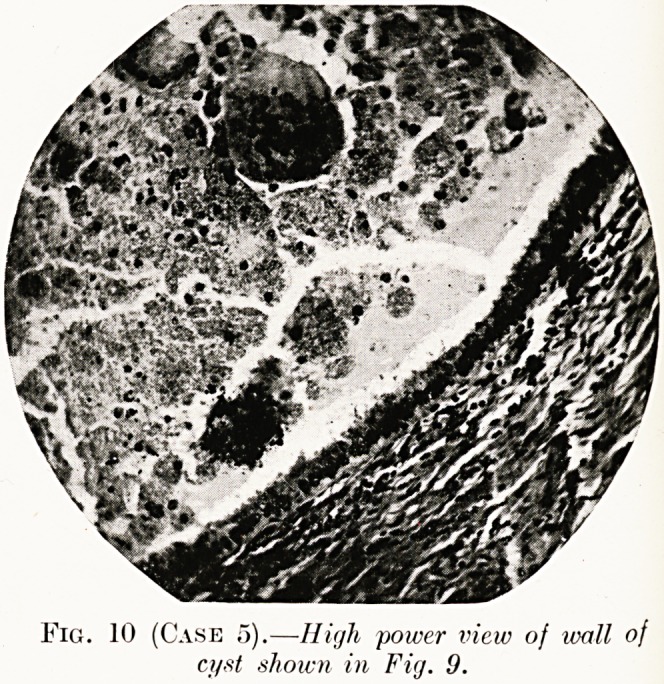


**Fig. 11 (Case 6). f11:**
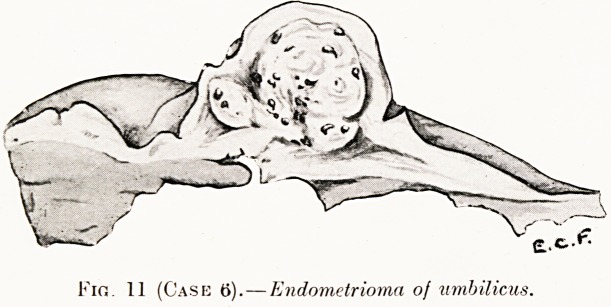


**Fig. 12 (Case 6). f12:**